# Microglia as a critical player in both developmental and late-life CNS pathologies

**DOI:** 10.1007/s00401-014-1321-z

**Published:** 2014-07-24

**Authors:** Noël C. Derecki, Natalie Katzmarski, Jonathan Kipnis, Melanie Meyer-Luehmann

**Affiliations:** 1 Department of Neuroscience and Center for Brain Immunology and Glia (BIG), School of Medicine, University of Virginia, Charlottesville, VA 22908 USA; 2 Department of Neurology, Neurocenter, University of Freiburg, 79106 Freiburg, Germany; 3 Faculty of Biology, Albert-Ludwigs-University of Freiburg, Freiburg, Germany

**Keywords:** Alzheimer’s disease, Rett syndrome, Mecp2, Phagocytosis, Amyloid plaques, Microglia, In vivo two-photon microscopy, Genome-wide association studies (GWAS)

## Abstract

Microglia, the tissue-resident macrophages of the brain, are attracting increasing attention as key players in brain homeostasis from development through aging. Recent works have highlighted new and unexpected roles for these once-enigmatic cells in both healthy central nervous system function and in diverse pathologies long thought to be primarily the result of neuronal malfunction. In this review, we have chosen to focus on Rett syndrome, which features early neurodevelopmental pathology, and Alzheimer’s disease, a disorder associated predominantly with aging. Interestingly, receptor-mediated microglial phagocytosis has emerged as a key function in both developmental and late-life brain pathologies. In a mouse model of Rett syndrome, bone marrow transplant and CNS engraftment of microglia-like cells were associated with surprising improvements in pathology—these benefits were abrogated by block of phagocytic function. In Alzheimer’s disease, large-scale genome-wide association studies have been brought to bear as a method of identifying previously unknown susceptibility genes, which highlight microglial receptors as promising novel targets for therapeutic modulation. Multi-photon in vivo microscopy has provided a method of directly visualizing the effects of manipulation of these target genes. Here, we review the latest findings and concepts emerging from the rapidly growing body of literature exemplified for Rett syndrome and late-onset, sporadic Alzheimer’s disease.

## Introduction

For decades, microglia have been conspicuously absent from most neuroscientific discussions regarding the critical players in the workings of the brain. If microglia were mentioned at all, it was more often than not in terms of their pathological role in neuroinflammation. Only very recently have these cells come under more careful consideration as to their unique place in the network of the central nervous system (CNS). As such, microglia have recently been revealed to be surprisingly more nimble and nuanced than previously imagined in their ability to respond to their surrounding environment. In the review that follows, we will discuss key studies highlighting the extraordinary roles these cells have now been shown to play in both CNS development and decline. In the development, we focus on the role of microglia in Rett syndrome; in decline, we look at microglia as major players in Alzheimer’s disease (AD) pathology.

## Microglia: the tissue-resident macrophages of the brain

Microglia are ubiquitous throughout the CNS, and are estimated to represent approximately 10 % of all cells found within the brain [70]. Microglia are unique within the brain in many ways, but perhaps primarily so in that they are true immune cells, originating during primitive hematopoiesis in the yolk sac as CX3CR1-expressing tissue-resident macrophage precursors [39]. Subsequently, precursors become migratory, move into the nascent brain, and proliferate in situ [1]. Recent lineage tracing experiments have suggested that expression of runt-related transcription factor (Runx)1, Pu.1 and Irf8 are critical, and that establishment of the brain vasculature is necessary for the infiltration of the CNS by these cells [39, 62, 63].

Cellular origin will frequently dictate function, and microglia share provenance with other tissue-resident macrophages [38, 45]. Thus, it can be expected that microglia should perform well as phagocytes, producers of cytokines, and of growth factors, as necessary [2]. Indeed, this has been shown to be the case. However, the nature of the tissue surrounding a cell will also impact upon its phenotype, and certainly microglia must ply their trade in immediate proximity to delicate neurons. Therefore, it is not surprising that molecules found in the CNS milieu act to modulate microglial function accordingly. TGF-β1, for instance, plays a major role in dampening microglial cytokine production, with loss having been shown to be associated with neuronal death and pathological microglial activation/microgliosis as measured at postnatal day 21 [15]. It is important to point out that the mouse model used succumbs to autoimmune-mediated lethality early in adulthood. In another recent work, TGF-β1^−/−^ mice in which T cell production of TGF-β is preserved, thus preventing the abovementioned peripheral autoimmune wasting phenotype, were shown to suffer from severe microglia deficiency, indicating that CNS tissue-resident macrophages and their surrounding tissue may engage in dynamic and mutual support [18]. The fact remains, however, that certain aspects of these two works are in conflict, and clearly more research is needed to better define the precise role for TGF-β in microglial function and survival. Fractalkine is another such modulatory molecule produced by neural cells, suggested to act directly on the microglial CX3CR1 receptor to prevent neurotoxicity [19, 71]. Thus, microglia are bona fide immune cells, but like other tissue-resident macrophages, they are also uniquely positioned to provide benefit and homeostatic support to the tissue within which they reside, in this case, the CNS. Microglia must be dynamic, but also judicious and measured in their responses. Moreover, microglial phenotype has been suggested to be plastic [104]. Yet, as guardians of the brain, they need to also possess the ability to respond vigorously to pathological insults when appropriate. Along these lines, recently developed imaging techniques have revealed that microglia are surprisingly dynamic cells, rather than inactive unless provoked, as was long believed [89].

## Microglia are active participants in developing brain

Microglia were long thought to be “quiescent” cells except during pathological insult; this notion, however, has lately been powerfully challenged by two-photon imaging studies indicating that microglia are in a state of perpetual surveillance even in healthy brain [89]. Resting microglia display highly ramified processes. Under physiological conditions, these processes are highly motile, constantly sampling their environment, while the soma remains stationary [28, 89]. In addition to performing active surveillance, microglia are also the professional phagocytes of the brain, and, as such, must be involved in unremitting cleanup of cellular corpses and debris. Phagocytosis of apoptotic cells is a necessary part of normal tissue development [101], and is thus integral to the role of microglia as tissue-resident macrophage. During the process of brain maturation, millions of neurons undergo apoptosis, yet leave barely a trace—thanks to vigilant corpse removal attributable largely to microglia [126]. Similarly, in experiments involving induced massive ablation of cells within the brain parenchyma, there is little—if any—detectable debris to be found even days after [95]. It is all the more striking that phagocytosis of apoptotic neurons is not merely a passive response. The active role of microglia in prompting apoptosis during development is well-illustrated by studies which demonstrate that deficiencies in microglial CD11b and DAP12, important in myeloid activation phenotype, were associated with impairment in hippocampal development [123].

Based on findings in these works and others, it is likely that impairment of normal microglial function—particularly during early life—could be expected to have serious implications regarding normal brain development. A dysregulated or deficient immune response could contribute significantly to pathologies seen in neurodevelopmental disorders involving abnormal dendritic arborization and dendritic spine maintenance. Along these lines, it was demonstrated in a series of studies that an interplay between several cell types in the CNS was ultimately responsible for the pruning of synapses by microglia in the visual system. Immune complement factors, namely C1q and C3, were shown to be expressed on neurons [113] as a result of TGF-β signaling by astrocytes [10] and recognized by cognate receptors expressed exclusively in brain parenchyma by microglia [103]. Pruning was suggested to be activity dependent [119] in works that correlated light deprivation and re-exposure with dynamic interactions between microglia and synapses [103] and directly by showing increased microglial phagocytosis of synaptic inputs corresponding to tetrodotoxin-mediated silencing compared to those activated by forskolin [103]. While these studies were conducted largely with a focus on the developing visual system, these results imply that similar microglial dysfunction in other brain areas could impact powerfully on synaptic maintenance and neural network activity, as is suggested to be important in autistic spectrum pathologies. A recently published work indeed suggests that network function and social behavior are impaired as a result of microglial deficiency and compromised microglial–neuron signaling [130].

## In vivo imaging of microglia in mouse models of AD

**Fig. 1:**
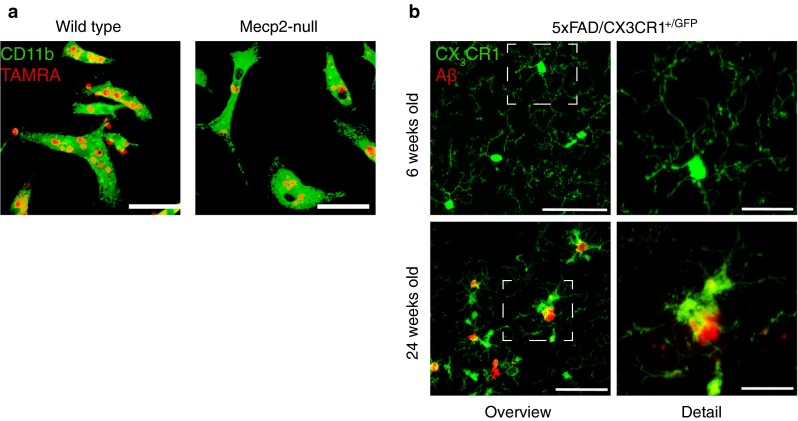
Aberrant microglia in models of Rett syndrome and Alzheimer’s disease. **a** Representative captures of phagocytosing microglia (labeled with anti-CD11b, green) incubated for 2 h with TAMRA-labeled UV-irradiated neural progenitor cells (*red*). **b** Representative confocal images of microglia in a pre-depositing 6-week-old 5xFAD transgenic mouse and activated microglia encompassing plaques in a 24-week-old 5xFAD transgenic mouse. Note the shortened and less ramified processes. *Green labeling* is GFP expressed under the control of the CX3CR1 promoter; red fluorescence depicts Aβ plaques labeled with an anti-Aβ antibody. *Scale bars*: **a** 25 µm and **b** left panel 50 µm; *right panel* 20 µm

The same methodology that has revealed the surprisingly dynamic role of microglia—two-photon microscopy [29]—has enabled in vivo long-term imaging studies and allowed monitoring of morphological and functional changes in the living mouse brain under normal and pathological conditions [72, 111]. A recent in vivo imaging study revealed that microglia cells undergo age-related morphological changes such as increased soma volume and a shortening of the processes [46]. Interestingly, aged mice exhibited increased microglial soma movement in comparison to younger mice, but this effect was diminished in response to acute injury by laser lesion [46]. Under pathological conditions, such as AD and in mouse models of AD, microglial cells are tightly associated with and cluster around dense core amyloid plaques [54], which are the major neuropathological hallmark of AD and are thought to be toxic to the surrounding neural tissue [17, 67, 82, 83, 112]. In terms of morphology, microglia display a reactive phenotype in AD with typically short, thickened and less ramified processes [54, 81, 116]. In the aged human brain, ‘dystrophic’ de-ramified microglia have been described with fragmented processes and bulbous swellings [115]. However, a similar dystrophic phenotype resembling morphological signs of aging has not yet been seen in the rodent brain. Instead, microglia in the rodent brain appear to be less complex [26, 46, 120]. In a mouse model of AD with plaque pathology, microglia had shorter processes (Fig. 1b) and less process movement compared to younger pre-depositing transgenic mice indicating, that age may impact the aforementioned morphological changes [65]. Nonetheless, the functional consequences of these morphological changes remain poorly understood and need further study.

Acute two-photon imaging identified that microglia cells surrounding Aβ plaques were morphologically mainly hypertrophic and amoeboid, whereas in plaque-free areas of the brain, ramified microglia were predominantly evident [14]. Ramified microglia were shown to rapidly migrate towards newly formed plaques [83], suggesting that they might transform into a hypertrophic or even amoeboid cell type in response to Aβ plaque formation and could have a pathological role. Recently, several in vivo imaging studies have monitored the appearance and growth of Aβ plaques [16, 47, 80, 83], which is of importance for the pathological development of AD. Plaque growth might be restricted by microglia removing and phagocytosing Aβ fibrils [11, 110]. This idea is supported by two immunization studies that provided in vivo evidence that microglia are able to phagocytose Aβ [4, 65]. The precise role of microglia in the pathogenesis of AD remains, however, poorly understood and the microglia response to plaques remains controversial. Surprisingly, microglial depletion in a mouse model of AD changed neither the plaque count nor the size of plaques [40]. However, microglia were only depleted for 4 weeks, and in older mice, which might explain the lack of effect.

In addition to plaques, AD is also characterized by massive neurodegeneration and neuronal cell loss, and several studies have tried to unravel how microglial activation might influence this neuronal loss. A combination of two-photon microscopy and laser ablation demonstrated that microglia cells adjacent to the site of injury polarized their processes towards the lesion to contain the injury [28]. In the context of AD, the neuronal cell loss in another triple transgenic mouse model of AD [91] was rescued after crossbreeding with mice lacking the CX3CR1 receptor, indicating a crucial role of the microglial chemokine receptor in mediating neuronal apoptosis in AD [36]. Other studies using CX3CR1-deficient mice showed exacerbated levels of phospho-Tau and elevated Tau pathology in Tau transgenic mice [9, 22], while CX3CR1 deficiency reduced amyloid load in AD mouse models with numerous Aβ deposits [71, 74]. Interestingly, neuronal and behavioral deficits worsened in CX3CR1-deficient mice and were plaque independent [22]. Despite these compelling results, the precise role of microglia in the pathogenesis of AD remains enigmatic. We predict two-photon microscopy will help shed light on the contribution of microglia to this devastating disease.

In the following sections, we take a look at evidence from genetic analyses that suggest a basis for immune abnormalities in Rett syndrome, previously considered to be a disease solely of neurons, and also summarize genetic approaches that have led to the recent discovery of a number of new AD risk and susceptibility factors including inflammation.

## Mecp2 as a regulator of immune-related genes

Rett syndrome is an X-linked CNS developmental disorder grouped with the autism spectrum disorders. Rett syndrome is linked in the majority of cases to mutations in the methyl-CpG-binding protein 2 (*MeCP2*) gene [3], which acts to modulate transcription at the level of chromatin remodeling by partnering with cofactors such as Sin3A in the formation of repressor complexes [21]. Interestingly, it was shown that Mecp2 functions as both activator and repressor of transcription, thereby complicating the predicted results of mutation of the gene. Regardless, mutation of *MeCP2* has been definitively linked to major disruptions of CNS and somatic function which together underly Rett syndrome pathology. Rett syndrome symptoms include psychomotor impairment, locomotor deficits, tremors, apneas, osteopenia, scoliosis, intestinal dysfunction and severe mental retardation [109]. *MeCP2* is an epigenetic regulator of thousands of downstream genes and is expressed in most cells, including at high levels in immune tissues [107]. While several investigations into genes dysregulated as a result of *Mecp2* deletion have been performed to date, the genes selected for further analysis have been those previously implicated in neuronal, rather than glial or immune function. However, microarray analyses of neural tissue from *Mecp2*-deficient mice have consistently included immune genes, e.g., Irak-1 [58], as among the most highly aberrant in transcription as a result of loss of *Mecp2*. Along these lines, *MeCP2* was recently named as a candidate susceptibility gene for systemic lupus erythematosus, an autoimmune disorder [124]. Nevertheless, research into immune system abnormalities in Rett syndrome has been surprisingly limited. In the few such studies conducted to date, however, it has become increasingly clear that mutations in *MeCP2* can greatly modify the expression of genes influencing the function of immune cells. Thus, it is not surprising that *Mecp2* has been directly connected to regulation of genes including *FoxP3*, a defining marker of T regulatory cells [68], and interferon (IFN)-γ, produced by several immune cell types including T lymphocytes and myeloid cells [118]. Notably, dysregulated IFN-γ production by T cells was recently suggested to be key to peripheral immune pathology common in *Mecp2* duplication syndrome, a disease similar in severity to Rett syndrome, but caused by overexpression of *Mecp2* rather than deficiency [129]. More recently, it was shown that *Mecp2* plays a role in the generation of T_H_1 and T_H_17 cells by the regulation of microRNA-124. This latest work is notable in that it elucidates a mode of gene regulation by *Mecp2* that is separate from its canonical function in chromatin modification [56].

Clinical data from Rett syndrome patients regarding immune function present a picture of overall dysregulation, but studies are few; thus, overarching patterns are still unclear. In general, the picture painted may be one of immune insufficiency rather than one implicating *Mecp2*-mutant immune cells as “bad actors”. For example, CD25-positive T lymphocytes were undetected in blood analyzed from Rett syndrome patients [98] indicating the possibility of activated effector T and T regulatory cell deficiency, both of which express CD25. Interestingly, an unrelated study showed increased serum levels of soluble CD25 [33] in serum from Rett syndrome patients. Increased soluble CD25 has been linked to both T regulatory and T effector cell repression. Additionally, a decrease in CD8^+^ T lymphocytes and natural killer cells has also been shown.

In sum, while several groups have focused on elucidation of downstream genetic targets of *Mecp2* in neurons or whole brain tissue, these data, while intriguing, have not as yet revealed many potential critical regulatory molecules that might serve as targets for manipulation. However, one that has shown some promise, first in mouse models [121] and now in Stage two clinical trials, is insulin-like growth factor (IGF)-1 [61]. It is perhaps noteworthy that microglia are a major producer of IGF-1 in the brain, thus supporting the notion of a central role for these immune cells in Rett syndrome pathobiology.

## GWAS studies for identifying risk genes in AD

Familial AD (FAD) is caused by specific, identifiable, autosomal dominant mutations in the Amyloid Precursor Protein (APP), Presenilin 1 and/or Presenilin 2 and is characterized by an early onset of the disease. However, the majority of AD patients suffer from sporadic AD, which has no definitive genetic cause. The etiology of sporadic AD is largely unknown, but environmental factors and genetic predisposition may be risk factors for the disease.

Recently, genome-wide association studies (GWAS) have emerged as an effective tool for identifying several new risk genes for Alzheimer’s disease. The most common risk factor for sporadic AD is the ApoE4 allele, which increases the risk of developing the disease by three times for heterozygous carriers and by 15 times for homozygous carriers [64]. Other genes, such as CLU, Bin1 or PICALM have also been identified as risk genes for AD [44, 52, 69, 105]. Among the genes identified as risk factors for AD were genes expressed by microglia. For example CD33 was identified as a sporadic AD risk locus [8, 50, 87]. CD33 is a transmembrane protein and a member of the sialic acid-binding immunoglobulin-like lectins (Siglecs). Its activity has been linked to triggering endocytosis and pathogen recognition, however, its function in the brain is still unknown [25]. Several new studies have recently examined the role of CD33 in AD. CD33 expression was found to be increased in circulating monocytes in carriers of the rs3865444^C^ risk allele and was associated with diminished internalization of Aβ peptide [13]. CD33 expression is also increased in the brains of AD patients and protein levels are specifically increased in the frontal cortex by twofold. Furthermore, CD33 expression in microglia is correlated with AD pathology, whereas the deletion of CD33 in a mouse model of AD lowered cortical and hippocampal Aβ plaque burden. Finally, in vitro experiments in BV2 cells with increased levels of CD33 diminished microglial uptake of Aβ, while the degradation was unaffected [41]. The authors implicated CD33 as a regulator of microglial clearance of Aβ and proposed it as a target for the treatment and prevention of AD.

Two recent reports linked another innate immune receptor and known microglial Aβ clearing molecule, triggering receptor expressed on myeloid 2 cells (TREM2) to increased risk of sporadic AD [42, 57]. TREM2 acts through DAP12 and, loss-of-function mutations lead to presenile dementia with bone cysts, also known as Nasu–Hakola disease [20, 94]. The function of this receptor in general and specifically its role in the pathogenesis of AD is not well understood. In the human and mouse cerebral cortex, TREM2 is predominantly expressed in microglia and to a much lesser amount in neurons [106]. TREM2 seems to be crucial for brain homeostasis, since its activation stimulates the phagocytic activity in microglia without causing inflammation [117]. In APP transgenic mice, TREM2 is up-regulated in microglial cells in the vicinity of amyloid plaques [34]. However, Nasu–Hakola disease patients do not overly develop Aβ plaques and therefore the relevance of TREM2 in sporadic AD needs to be investigated further.

Interestingly, CD33 and TREM2 both were found to be linked to DAP12 [131]. As aforementioned, DAP12 is crucial for normal brain development and for the clearance of apoptotic neurons. The authors used an integrated systems approach and ranked network structures for their relevance in sporadic AD. DAP12 was identified as being in the center of this microglial network and unifying previous top GWAS risk loci including CD33.

In the final sections, we suggest immune approaches aimed at the possible amelioration of Rett syndrome and Alzheimer’s disease, and highlight some critical functions of microglia, that, when impaired, may provide clues that link these seemingly disparate brain pathologies.

## Immune-directed treatments in Rett syndrome

While MeCP2 is highly expressed in neurons, it is also expressed in many other cells and tissues, including microglia [78] (Kipnis Lab, personal communication). Rett pathology was originally believed to be solely due to alteration or loss of *Mecp2* expression in neurons; however, more recent data suggest that glia also play a major role in the disease [6, 78]. For example, expression of wild-type *Mecp2* in astrocytes of otherwise *Mecp2*-null mice was shown to significantly block disease progression [73]. It was also proposed that *Mecp2*-null microglia might be directly damaging neuronal dendrites via supranormal production of glutamate [78]. In parallel, our analysis of primary microglia revealed a striking impairment of phagocytic capability by *Mecp2*-null microglia as compared to controls (Fig. 1a). Subsequent examination of brain tissue revealed increased levels of debris in *Mecp2*-null mice, suggesting that insufficient clearance of debris by *Mecp2*-null microglia could be a key factor contributing to Rett pathology [30].

If intrinsic impairment of microglia as a result of *Mecp2* deficiency were important to pathology, then supplementation of the CNS with wild-type microglia might be expected to yield benefit. A strategy of bone marrow transplant was previously shown by several groups to result in engraftment of microglia-like cells within brain parenchyma [84, 99] and such methods have been employed with some modest success in human disease in the treatment of globoid cell leukodystrophy (Krabbe disease) and related metabolic storage diseases associated with CNS pathology [51, 66]. Murine studies using the twitcher mouse model for Krabbe disease preceded clinical therapies, and showed CNS and peripheral engraftment followed by significant remyelination [51].

Indeed, *Mecp2*-null mice that received bone marrow transplant displayed remarkably blunted pathology. Although still different from wild-type mice, treated mice significantly outlived both *Mecp2*-null mice transplanted with *Mecp2*-null bone marrow and untreated controls. When brains from transplanted mice were examined, robust engraftment of microglia-like cells in brain parenchyma was observed, suggesting that arrest was associated with improved ability of wild-type microglia to support *Mecp2*-null neurons. Results from bone marrow transplant were supported by results from a complementary genetic/pharmacological approach using *Lysm* ^*cre*^ *Mecp2* ^*lox*-stop/y^ mice, in which arrest was achieved wild-type *Mecp2* was driven in a significant proportion of myeloid cells, including microglia, on an otherwise *Mecp2*-null background. These mice were treated with chronic injections of annexin V, shown to be effective in binding to phosphatidylserine residues exposed on apoptotic cells and critical for corpse recognition and removal. As expected, blocking recognition and uptake of debris in these mice by annexin administration resulted in abolishment of disease arrest and significant increase in TUNEL-positive debris in brains. These data strongly suggested that arrest was being mediated in otherwise *Mecp2*-null mice by the ability of wild-type microglia to effectively clear debris in CNS.

A further possibility is a scenario in which microglia may also be deficient in production of growth factors needed to maintain neural cells. Along these lines, it was recently shown that acute ablation of microglia or ablation of BDNF production by microglia resulted in deficits in dendritic spine elimination and behavioral impairments in motor learning, fear conditioning, and novel object recognition [95]. Similarly, mice deficient in hematopoietic-specific BDNF production exhibited pathological levels of hyperphagia and insulin resistance. IBA1-positive cells in brain parenchyma of these animals were visualized in hypothalamus in contact with neurons that are shown to control feeding. Importantly, BDNF-competent cells engrafted into hypothalamus following intracerebroventricular injection were associated with reversal of pathological feeding phenotype [122].

These works and others suggest the strong possibility that pathologies conventionally considered to be the result of isolated neural dysfunction may in fact be influenced significantly—and even caused in some cases—by microglial dysfunction. Along these lines, recent studies now highlight a central role for microglia, and more specifically microglial receptors in Alzheimer’s disease, a pathology classically associated with aging brain, but long assumed to be primarily of neuron-intrinsic etiology.

## Immune-receptors on microglia as therapeutic target for the treatment of AD

**Fig. 2:**
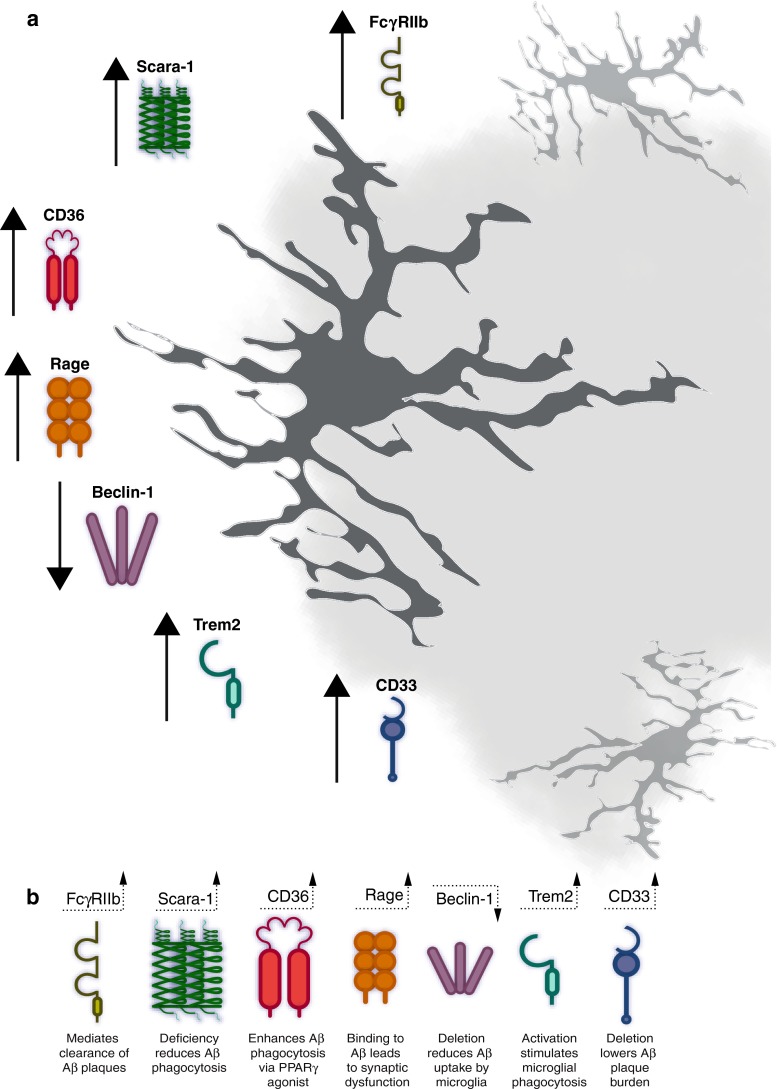
Regulation of microglial phagocytosis via receptors. **a**, **b** Summary of known receptors expressed on microglia and an autophagic protein that are involved in the phagocytosis of Aβ and are described in the context of AD pathology. The *faint gray shadow* in the background represents a plaque surrounded by several microglia. *Arrows* indicate increased or decreased receptor expression in AD. *Receptor shapes* are not meant to represent actual receptor structures

Microglia are the major phagocytic cells in the brain which become activated upon contact with Aβ [81]. To date, compelling evidence is lacking as to whether microglia are truly able to phagocytose Aβ [100]. This occurrence would not only involve the engulfment of the protein, but also a degradation step (degradation of Aβ), which has yet to be proven. Without the capability to degrade Aβ, it seems unlikely that amyloid-containing microglia can clear additional Aβ but instead may even transform into toxic cells. Nevertheless, growing evidence suggests that microglia are able to prevent or decelerate AD by promoting the clearance of Aβ. Several microglial receptors seem to play a pivotal role in the clearance of Aβ (Fig. 2). One of these receptors involved in the clearance of Aβ is the scavenger receptor expressed on microglia Scara-1. Scara-1 promotes the binding and phagocytosis of Aβ in vitro [31], and in vivo in a mouse model of AD [35]. Isolated human microglia also bind Aβ via Scara-1 receptor [53]. Furthermore, microglia that decorate Aβ plaques in a mouse model of AD showed increased levels of Scara-1 [12], whereas microglia isolated from Scara-1 knockout mice had reduced Aβ clearance capacity compared to wild-type cells [23]. Scara-1 deficiency increased Aβ plaque pathology in APPPS1 transgenic mice and accelerated the disease whereas pharmacological up-regulation of Scara-1 on mononuclear phagocytes had the opposite effect and increased clearance of Aβ [35] emphasizing the relevance of Scara-1 in the pathogenesis of AD.

Another microglial receptor for Aβ of the scavenger receptor family is CD36. CD36 binds Aβ fibrils and is expressed on microglia in the AD brain [24]. Additionally, CD36 mediates reactive oxygen species production in response to Aβ [24] and microglial recruitment to the site of aggregated fibrillar Aβ in vivo [32]. Investigation of the signaling molecules of microglial CD36 upon Aβ stimulation helped to identify Src family members such as Fyn and Lyn as well as MAPK [85]. There have been attempts to increase the phagocytic activity of microglia via peroxisome proliferator-activated receptor γ (PPARγ) agonists that reduced Aβ plaque burden in APP transgenic mice. This enhancement of microglial Aβ phagocytosis was shown to be mediated by the CD36 receptor [127]. Interestingly, CD36 is a co-receptor as it can form a heterodimeric complex with TLR-4 and TLR-6. The interaction of Aβ with this receptor complex on microglia provokes an inflammatory response with IL-1β production indicative of inflammasome activation [114].

This is well in line with the finding that microglial Beclin-1 is also impaired in AD and regulates phagocytic receptor function. Beclin-1 is known for its role in the autophagosomal system, which is in turn implicated in AD. Early in AD progression, Beclin-1 expression and protein levels in the human enthorinal cortex decrease [97]. A heterozygous deletion of Beclin-1 in a mouse model for AD increased the accumulation of intraneuronal and extracellular Aβ, most likely due to the accumulation of APP and its metabolites [55, 97]. Beclin-1 is important for efficient phagocytosis by microglia in vitro and in vivo. Reduced Beclin-1 expression also caused microglial changes; microglia and microglia-like cell culture lines deficient for Beclin-1 were only able to clear a minor amount of Aβ aggregates [75]. The author further claims that together with its binding partner Vps34, Beclin-1 regulates a retromer complex which is among others involved in receptor recycling. Interestingly, reduced Beclin-1 levels also diminished phagocytic receptor recycling for TREM2 and CD36, which both are linked to microglial impairment in AD and are discussed as well in this review.

Another microglial receptor reported to bind to Aβ is FcγRIIb. FcγRIIb is expressed on most leucocytes excluding natural killer cells. It contains an ITIM in its cytoplasmic domain which recruits phosphatases in turn resulting in an inhibition of cell activation (for review see [92]). Like other FcRs, FcγRIIb is widely expressed in the central nervous system, and is important for cerebral function and development, especially for Purkinje cells [88]. However, its role in AD still remains elusive. The first hint came from a study by Peress et al. 1993, identifying FcγR immunoreactivity on microglia and in senile plaques distributed throughout the white matter and cortex in healthy and AD brains [96]. This was further extended and confirmed with the finding that FcγRIIb was specifically up-regulated in the hippocampus of AD brains [59]. From immunization studies, there is evidence for FcγRIIb-mediated phagocytosis by microglia that increases the clearance of Aβ plaques in vivo [5, 7]. However, another immunization study using FcγR knockout mice did not conclude that FcγRIIb-mediated phagocytosis was the crucial phagocytic mechanism [27]. A recent paper linked Aβ oligomers and FcγIIb receptor binding as a possible cause of neurotoxicity by activating caspase-12 and boosting endoplasmic reticulum stress markers [59]. Furthermore, oligomer-specific antibodies to Aβ lead to increased neurotoxicity of Aβ in a mixed culture of primary neuronal and glial cells. Both the depletion of the Fc region and the removal of microglia from the culture diminished this effect implicating microglia as a crucial mediator of neuronal death [86]. Inhibition of the Aβ–FcyR interaction could be a new therapeutic approach for AD and might bring a revival of immunization trials.

Yet another example of a receptor involved in the pathogenesis of AD is the receptor of advanced glycation end products (RAGE) that has also been shown to bind to Aβ [128]. RAGE is expressed in multiple cell types, including microglia and is up-regulated in hippocampal microglia of AD brains (see review by [77]). The interaction of RAGE with Aβ has diverse implications on inflammatory responses, neuronal function and the elevation of amyloidosis. Microglial activation elicited from the binding of Aβ to RAGE produced cytokines such as IL-1β and TNF-α, which in turn might lead to the clustering of microglia around Aβ plaques [76, 128]. Furthermore, microglial RAGE signaling through p38MAPK and JNK released IL-1β, leading to synaptic dysfunction [93]. In a mouse model for systemic amyloidosis, the use of an anti-RAGE antibody significantly reduced amyloid plaque formation. Although promising results have been achieved with the RAGE inhibitor PF-04494700 from Pfizer in preclinical studies in transgenic mice and in a 10-week Phase 2 trial [102], the follow-up Phase 2 trial over 18 months was halted because of serious side effects and worsened cognitive decline in the higher dose treatment group. In contrast, the lower dose group did not raise safety concerns and some analyses showed decreased decline on the Alzheimer’s Disease Assessment Scale-cognitive (ADAS-cog), but other clinical and biomarker measures failed to show significant differences between low-dose and placebo group (for information see http://clinicaltrials.gov) [37].

## Developmental and age-related CNS pathologies: is microglia dysfunction a common element?

While Rett syndrome and Alzheimer’s disease are clearly different in terms of time of age of onset and pathological sequelae, in terms of microglial dysfunction as a contributing factor, they may indeed have much in common. As was detailed previously, in a mouse model of Rett syndrome, data suggest that the ability of microglia to respond efficiently to a buildup of apoptotic corpses during development may be one of the many keys to the postnatal onset of pathology. Phagocytosis of apoptotic neurons has long been recognized as necessary to normal brain development, and more recently, it is suggested that microglia also play a critical role in the synaptic pruning process.

Similarly, as covered earlier in this review, GWAS studies and other genetic screening techniques have identified microglial receptors as strongly up- or down-regulated in the Alzheimer’s disease brain. For instance, during the progression of AD, the expression of several Aβ binding receptors such as the Scara-1, CD36 and RAGE decreased by two- to fivefold in microglia, and thereby lost their ability to clear Aβ [49]. Taken together, these studies provide the basic rationale for the development of further therapeutic strategies targeting microglia, e.g., enhancing the ability of microglia to clear Aβ by an up-regulation of these microglial receptors (Fig. 2).

Dysregulated inflammatory response may also represent a common mechanism by which microglial impairment contributes to pathology in both diseases. *Mecp2* is a known regulator of NFκB though multiple pathways, including via the aforementioned Irak1, and also through direct regulation of the IκBα promoter [79]. Along these lines, it was recently shown that loss of *Mecp2* in immune cells, including neonatal mouse microglia and siRNA-treated human monocytes, led to supranormal production of glutaminase via an NFκB-dependent process [90]. Glutamate is well-recognized as a factor in neurotoxicity, and unrestrained production within the confines of the CNS by microglia would conceivably lead to adverse effects on neuronal function. The possibility of dendritic and synaptic damage mediated by microglia-derived glutamate was previously proposed as an underlying factor in Rett syndrome pathology [78].

In the context of AD, the NLRP3 inflammasome pathway in microglia has been elucidated over the last few years as possibly being of critical importance. Fibrillar Aβ was shown to activate the NLRP3 inflammasome with IL-1β secretion by microglia [43]. Interestingly, inhibition of phagocytosis reversed this effect and resulted in reduced NLRP3-mediated IL-1β release [43]. As mentioned above, CD36 is able to bind to Aβ [125], and its uptake was shown to promote NLRP3 activation and amyloid aggregation at least in cell culture experiments [108]. More in vivo evidence for the involvement of the NLRP3 inflammasome pathway in the pathogenesis of AD came recently from a study by Heneka et al. which clearly demonstrated an increase in caspase-1 activation in diseased human AD brains. Furthermore, NLRP3 deficiency in APP transgenic mice decreased Aβ plaque load, suggesting a prominent role of NLRP3 in the pathogenesis of AD and a potential target for therapeutic intervention [48].

## Conclusion

The long-held view that microglia are at best inactive unless provoked by pathogens, and at worst “the enemy within” [60] has recently been challenged by several high-profile studies. Lineage tracing and genetic analysis have revealed that microglia share origins with other tissue-resident macrophages [38, 39], thus suggesting an important homeostatic role in tissue maintenance, a trademark function of resident macrophages. In support of this notion, in vivo imaging has revealed, that microglia are indeed dynamic surveyors of the brain’s milieu, even during health [89]. During development, microglia are now shown to be necessary for pruning of supernumerary synapses and clearance of apoptotic neurons [103, 119]. Accordingly, deficiencies in phagocytic function may be linked to autistic spectrum disorders, which frequently present with dendritic pruning defects. In the aging brain, microglia have been similarly implicated as mediators of both healthy function and pathology. Aged microglia have been demonstrated to display an amoeboid morphology in particular when associated with Aβ plaques [14], a classical hallmark of AD pathology. Along these lines, it has become increasingly clear that expression of key receptors on microglia may be linked to their response to Aβ, shifting them to either ameliorative or aggressive phenotype.

Thus, the dogmatic view of microglial response solely as an indicator of pathology is becoming revised as it becomes clear that microglial inactivity is as dangerous as overreaction. We should seek to encourage a robust and well-tuned response from microglia, rather than a diminished response, as necessary for maintained CNS health.
